# Factors associated with life-sustaining treatment restriction in a general intensive care unit

**DOI:** 10.1371/journal.pone.0181312

**Published:** 2017-07-18

**Authors:** Stein Arve Skjaker, Henrik Hoel, Vegard Dahl, Knut Stavem

**Affiliations:** 1 Section of Orthopaedic Emergency, Division of Orthopaedic Surgery, Oslo University Hospital, Oslo, Norway; 2 Department of Surgery, Sykehuset Innlandet Kongsvinger, Kongsvinger, Norway; 3 Department of Anaesthesiology, Surgical Division, Akershus University Hospital, Lørenskog, Norway; 4 Institute of Clinical Medicine, University of Oslo, Oslo, Norway; 5 Department of Pulmonary Medicine, Medical Division, Akershus University Hospital, Lørenskog, Norway; 6 Health Services Research Unit, Akershus University Hospital, Lørenskog, Norway; National Yang-Ming University, TAIWAN

## Abstract

**Purpose:**

Few previous studies have investigated associations between clinical variables available after 24 hours in the intensive care unit (ICU), including the Charlson Comorbidity Index (CCI), and decisions to restrict life-sustaining treatment. The aim of this study was to identify factors associated with the life-sustaining treatment restriction and to explore if CCI contributes to explaining decisions to restrict life-sustaining treatment in the ICU at a university hospital in Norway from 2007 to 2009.

**Methods:**

Patients’ Simplified Acute Physiology Score II (SAPS II), age, sex, type of admission, and length of hospital stay prior to being admitted to the unit were recorded. We retrospectively registered the CCI for all patients based on the medical records prior to the index stay. A multivariable logistic regression analysis was used to assess factors associated with treatment restriction during the ICU stay.

**Results:**

We included 936 patients, comprising 685 (73%) medical, 204 (22%) unscheduled and 47 (5%) scheduled surgical patients. Treatment restriction was experienced by 241 (26%) patients during their ICU stay. The variables that were significantly associated with treatment restriction in multivariable analysis were older age (odds ratio [OR] = 1.48 per 10 years, 95% confidence interval [CI] = 1.28–1.72 per 10 years), higher SAPS II (OR = 1.05, 95% CI = 1.04–1.07) and CCI values relative to the reference of CCI = 0: CCI = 2 (OR = 2.08, 95% CI = 1.20–3.61) and CCI≥3 (OR = 2.72, 95% CI = 1.65–4.47).

**Conclusions:**

In multivariable analysis, older age, greater illness severity after 24 h in the ICU and greater comorbidity at hospital admission were independently associated with subsequent life-sustaining treatment restriction. The CCI score contributed additional information independent of the SAPS II illness severity rating.

## Introduction

Increasing therapeutic possibilities have made decisions to withhold or withdraw life-sustaining treatments in intensive care units (ICUs) more common [1–3]. Many patients who have treatment withheld or withdrawn die during their hospital stay [4], and most of the deaths in the ICU occur after such decisions are taken [3, 5, 6]. It is therefore important to understand if information available during the initial period of the ICU stay is associated with later decisions to withhold or withdraw life-sustaining treatment.

Comorbidity is common among ICU patients and may influence their acute illnesses, types and intensity of care, and treatment outcome. It is also an important determinant of ICU mortality [7–10]. Patient comorbidity needs to be considered when assessing whether treatment should be withheld or withdrawn. This is, however, rarely assessed using an aggregated index. Comorbidity can be assessed using the Charlson Comorbidity Index (CCI), which was developed to classify comorbidity and to estimate risk of death from comorbid diseases [11]. No previous studies have investigated the relationship between comorbidity as assessed using CCI scores and the decision to withhold or withdraw life-sustaining treatment in an ICU.

Some studies have investigated factors associated with the life-sustaining treatment restriction in specific patient groups, such as trauma patients [12], neurological patients [13], surgical patients [14, 15], patients receiving haemodialysis [16], patients undergoing cardiac arrest [17], or patients with advanced chronic obstructive pulmonary disease [18]. A British multicentre study found that older age, a history of one or more severe medical conditions, emergency surgery, medical admission, cardiopulmonary resuscitation within 24 h prior to ICU admission, mechanical ventilation during the first 24 h in the ICU, and sedation and/or paralysis were independently associated with the decision to withdraw active treatment [19]. That study recorded comorbid conditions, but they were not weighted according to their severity or number.

According to Norwegian national guidelines, decisions regarding the withholding or withdrawing of life-sustaining treatment should be based on medical judgments, taking the patient’s or next of kin’s view into consideration, and the decision should be documented in the patient’s medical record [20]. However, this is often insufficiently documented in the medical record [21], and factors other than those stated may have influenced the decisions.

The aims of this study from a general ICU in Norway, were (1) to identify factors associated with the life-sustaining treatment restriction, defined as having treatment either withheld or withdrawn, and (2) to explore if CCI scores contributed to explain decisions to restrict life-sustaining treatment.

## Materials and methods

### Population and data collection

**Fig 1:**
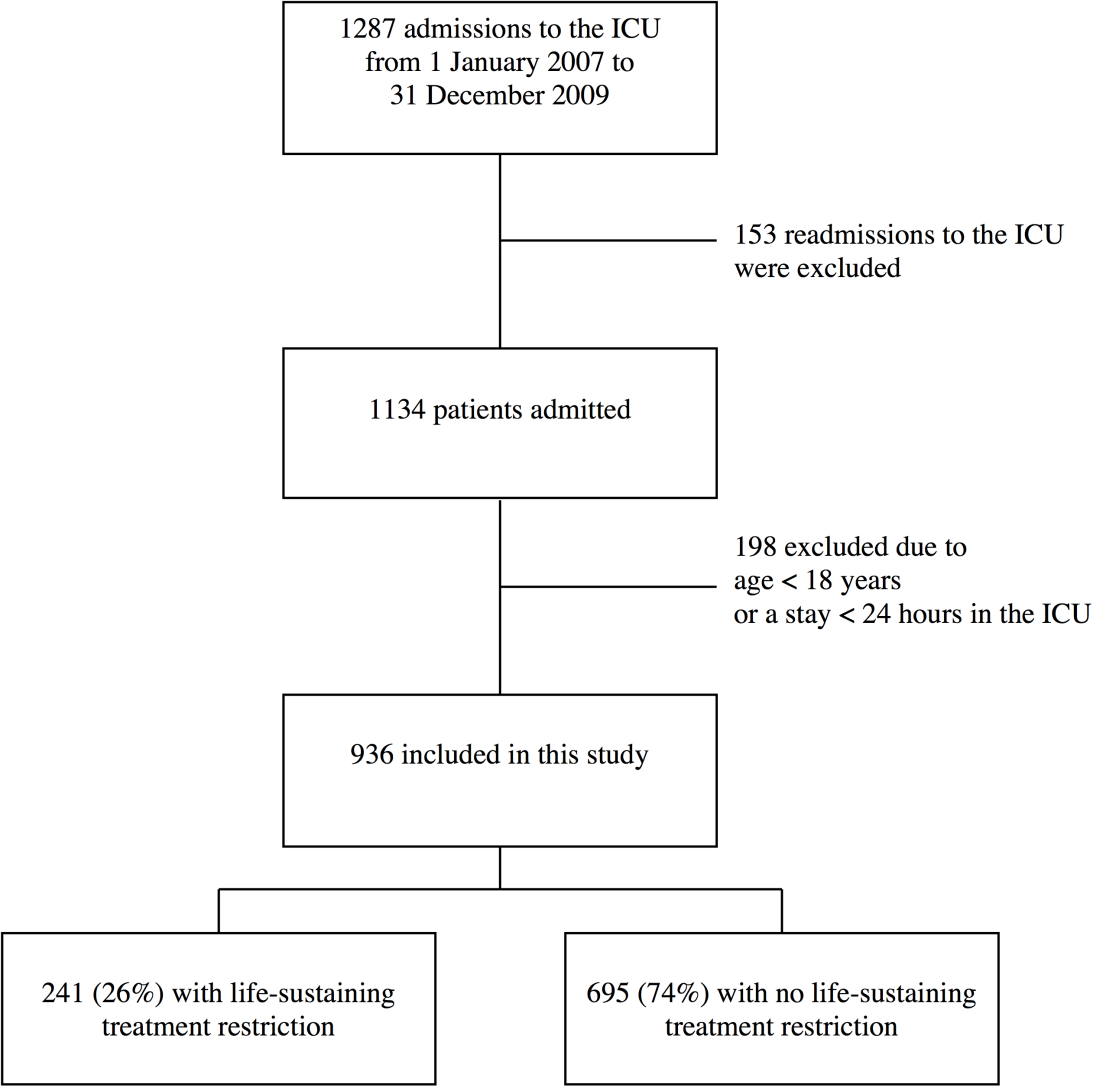
Flow chart of patients included in the study from the intensive care unit.

Akershus University Hospital had a catchment population of about 320,000 people in Oslo and Akershus, Norway at the time of this study. The hospital had a nine-bed general ICU, which treated patients from medical, surgical and neurological departments. The hospital had no program for cardiac surgery or neurosurgery. There were 1,287 patient admissions in this ICU between 1 January 2007 and 31 December 2009. After excluding 351 patients aged <18 years, an ICU stay of <24 h, or ICU readmission, we included 936 patients with their first admission to the ICU during this 3-year period (Fig 1).

The electronic medical records of all patients were retrospectivly reviewed by one of the authors (H.H. or S.A.S.), who recorded the patients’ comorbidity scores according to CCI criteria [11]. The two assessors used a standard registration form during the chart review.

Withholding treatment was defined as the decision not to start or increase a life-sustaining intervention, and withdrawing treatment was defined as the decision to actively stop a life-sustaining intervention [22]. Treatment restriction was defined as having treatment either withheld or withdrawn.

### Variables

We chose to collect information on variables that could be associated with treatment restriction on the basis of previous studies from ICUs with similar outcomes [15, 23] and studies using mortality as outcome [24–28], which we thought would be related to the outcomes in the present study. The variables are listed below.

Patients were assessed using the Simplified Acute Physiology Score II (SAPS II) during the first 24 h in the ICU [28]. SAPS II represents an illness severity rating for ICU patients that is based on the worst values measured for 17 variables during the initial 24 h of their ICU stay. It is scored on a scale from 0 to 163 points, with a higher score indicating a greater illness severity. In addition, a mathematical formula is provided to predict hospital mortality.

The SAPS II results were stored in a local database together with information on age, sex, type of admission, outcome of stay, time on mechanical ventilation, and whether treatment was withheld or withdrawn. Patients were grouped into the following eight principal disease categories based on the main disease categories in the Acute Physiology, Age, and Chronic Health Evaluation III (APACHE III) prognostic system: respiratory, cardiovascular/vascular, gastrointestinal, neurological, sepsis, trauma, metabolic, and others [24]. The dates and times of hospital admission, ICU admission and discharge were extracted from the patient administrative system of the hospital in order to calculate the length of the hospital stay prior to ICU admission and the length of the ICU stay.

We defined the type of admission as medical (n = 685; patients who did not receive surgery within 1 week prior to ICU admission), scheduled surgical (n = 47; patients whose surgery was scheduled at least 24 h in advance) or unscheduled surgical (n = 204; patients added to operating room schedule within 24 h of their surgery), according to the type of admission in the SAPS II system[28]. In the model, we included only variables available after the first 24 h of the ICU stay and did not subsequently update any of the variables.

Further details on data collection, the process of withholding or restricting treatments, and which life-sustaining treatments were impacted have previously been presented [21].

### Data analysis

We present descriptive statistics using mean and standard deviation values, median and 25th and 75th percentile values, or number (%) values. Groups were compared using the *t*-test for normally distributed data and the Mann-Whitney U test for non-normally distributed data. Categorical data were compared using the chi-square test or Fisher’s exact test, as appropriate.

We performed univariable and multivariable logistic regression analyses with the treatment restriction as the dependent variable, and selected available independent variables based on information from the literature. In the multivariable analysis we initially entered all variables for which p<0.25 in the univariable analysis, and then removed variables one by one, retaining sex and variables for which p<0.05 in the model [29]. For the CCI, we chose to use the score indicating normal (CCI = 0) as the reference, while the largest groups were chosen as references for the principal disease category and type of admission. The importance of CCI levels as a variable in the model was tested using a likelihood ratio test. Any problems with multicollinearity were identified by assessing Spearmans’s rank correlation between the independent variables in the multivariable model, which indicated that the coefficients for all pairwise correlations were <0.45. We used Stata (version 14.1, StataCorp, College Station, TX, USA) for all of the data analyses.

### Ethics

The study was based on routine data collected in the hospital. It was approved by the local privacy ombudsman for research at Akershus University Hospital. The study was also presented to the Regional Committee for Medical Health Research Ethics, which assessed the project as a quality assurance project (Ref: 2011/950), and no informed consent was required.

## Results

### Characteristics of patients

This study included 936 patients who were treated in the ICU from 2007 to 2009, of which 58% were men. A total of 241 patients (26%) experienced life-sustaining treatment restriction during their ICU stay. The most common principal disease categories upon ICU admission were respiratory (26%), cardiovascular/vascular (16%), gastrointestinal (15%) and neurological (10%). The distribution of principal disease categories differed between the patients who had treatment withheld or withdrawn and the patients without treatment restriction (p<0.001).

Patients who experienced treatment restriction were older (mean age 70 versus 55 years), had a greater illness severity (mean SAPS II score 55 versus 38 points), had higher estimated hospital mortality (mean 0.54 versus 0.27), had more severe comorbidity (all p<0.001), and had a longer hospital stay prior to ICU admission (median 0.6 vs. 0.3 days, p = 0.001) compared with patients who received full active treatment (Table 1). Treatment restrictions did not differ between males and females.

**Table 1:** Characteristics of patients and outcomes of treatment, number (%) unless otherwise stated.

	Total	Treatment withheld/withdrawn	No treatment restriction	p
n	936	241	695	
Age, years, mean ± standard deviation (SD)	58.8 ± 18.1	70.2 ± 12.6	54.9 ± 18.1	<0.001
Male sex	540 (58)	144 (60)	396 (57)	0.45
Simplified Acute Physiology Score II, mean ± SD^a^	42.5 ± 17.1	54.8 ± 16.7	38.3 ± 15.1	<0.001
Estimated mortality, mean ± SD	0.342 ± 0.269	0.539 ± 0.273	0.274 ± 0.232	<0.001
Charlson Comorbidity Index score				<0.001
0	352 (38)	39 (16)	313 (45)	
1	199 (21)	49 (20)	150 (22)	
2	153 (16)	52 (22)	101 (14)	
3	232 (25)	101 (42)	131 (19)	
Time to intensive care unit, days^b^	0.3 (0.1–2.6)	0.6 (0.1–4.6)	0.3 (0.1–2.0)	0.001
Length of intensive care unit stay, days^b^	2.7 (1.0–7.0)	2.7 (1.2–8.0)	2.7 (0.9–6.9)	0.099
Time on mechanical ventilation, days^b^	1.3 (0.1–5.3)	1.9 (0.7–7.2)	0.9 (0.0–4.8)	<0.001
Died in the intensive care unit	155 (17)	125 (52)	30 (4)	<0.001
Died before hospital discharge	262 (28)	187 (78)	75 (11)	<0.001
Principal disease category^c^				<0.001
Respiratory	248 (26)	64 (27)	184 (26)	
Cardiovascular/vascular	153 (16)	71 (29)	82 (12)	
Gastrointestinal	143 (15)	42 (17)	101 (15)	
Neurological	93 (10)	22 (9)	71 (10)	
Sepsis	70 (7)	20 (8)	50 (7)	
Trauma	52 (6)	8 (3)	44 (6)	
Metabolic	98 (10)	4 (2)	94 (14)	
Other	79 (8)	10 (4)	69 (10)	
Type of admission^d^				0.21
Medical	685 (73)	182 (76)	503 (72)	
Unscheduled surgical	204 (22)	52 (22)	152 (22)	
Scheduled surgical	47 (5)	7 (3)	40 (6)	

^a^ Higher score indicates more severe illness.

^b^ Data presented as median (25th-75th percentile).

^c^ Based on major disease categories in Acute Physiology, and Chronic Health Evaluation III (APACHE III) prognostic system.

^d^ Type of admission according to Simplified Acute Physiology Score.

The overall ICU and hospital mortality rates were 17% and 28%, respectively. The hospital mortality rate differed significantly between patients with treatment restriction (78%, 187 of 241) and patients without treatment restriction (11%, 75 of 695; p<0.001). Treatment was restricted in 187 (71%) of the 262 patients who died during the hospital stay and in 125 (81%) of the 155 patients who died in the ICU. None of the patients survived their ICU stay after withdrawal of any kind of life-sustaining treatment.

### Determinants of the life-sustaining treatment restriction

In univariable analysis, older age, higher SAPS II, and higher CCI produced higher odds of having treatment restriction.

**Fig 2:**
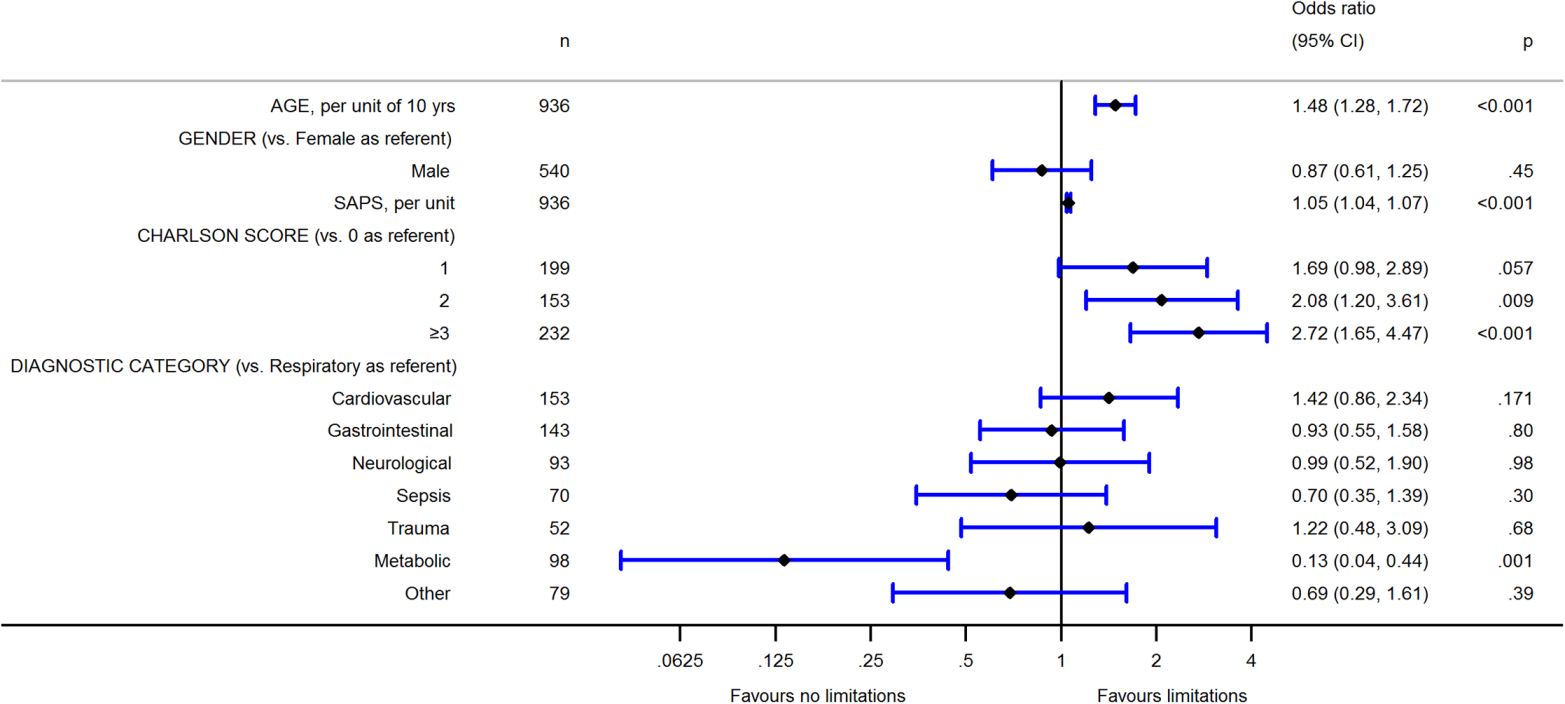
Factors associated with the life-sustaining treatment restriction, multivariable logistic regression analysis.

The independent variables in the multivariable model that were associated with the life-sustaining treatment restriction were older age (odds ratio [OR] = 1.48 per 10 years, 95% confidence interval [CI] = 1.28–1.72, p<0.001), higher SAPS II (OR = 1.05, 95% CI = 1.04–1.07, p<0.001) and the following CCI values relative to the reference of CCI = 0: CCI = 2 (OR = 2.08, 95% CI = 1.20–3.61, p = 0.009) and CCI≥3 (OR = 2.72, 95% CI = 1.65–4.47, p<0.001) (Fig 2). The addition of CCI levels as a variable in the model was statistically significant, as assessed with the likelihood-ratio test (chi^2^ (3) = 16.58, p<0.001). Among principal APACHE III disease categories, the metabolic category had lower odds for life-sustaining treatment restriction (OR = 0.13, 95% CI = 0.04–0.44, p = 0.001) compared to the reference respiratory category. Sex was not associated with treatment restriction.

## Discussion

In this study, patients were more likely to experience treatment restriction if they were older, had a more severe acute illness at ICU admission, or had more severe comorbidity as assessed by CCI at hospital admission. Also, patients with a metabolic principal disease category were less likely to experience treatment restriction during their ICU stay.

The finding that age was an independent predictor of treatment restriction is consistent with the literature [22, 30–33], and may indicate that the oldest patients receive less treatment [34, 35]. Older critically ill patients are also more likely to have new treatment restrictions independent of their baseline status, ICU-related illness severity, and worse organ dysfunction status [36].

SAPS II provides an estimate of the severity of the illness and the risk of death without having any primary diagnosis [28]. In the present study, SAPS II was independently associated with the decision to withhold or withdraw treatment. This supports previous studies using similar outcomes [15, 23]. The findings are also in line with SAPS II studies using mortality as an outcome [25, 26].

Patients with a metabolic principal disease category had lower odds for having treatment restriction in this study. This may be explained by a high proportion of patients with intoxication and diabetic ketoacidosis who were included in this category. These patients would have a favorable prognosis and an expectation of rapid improvement if given appropriate treatment.

In this study the CCI score was independently associated with treatment restriction, and hence, the score provided information in addition to that provided by the SAPS II. Several studies involving various populations, including ICU patients, have found that higher CCI scores are associated with increased mortality [8, 27]. Some studies have found specific chronic illnesses such as malignancy, chronic heart failure, chronic respiratory insufficiency, chronic neurological disease and severe cerebral motor disability to be associated with life-sustaining treatment restriction [7, 37, 38].

We are not aware of any previous studies that have shown comorbidity assessed with the CCI scores to be an independent factor associated with life-sustaining treatment restriction in the ICU. The CCI was originally developed for assessing the 1-year mortality of patients in a medical department [11], and hence, a scoring system that is more specific to the comorbidities experienced by ICU patients and a shorter time perspective might be more appropriate.

The incidence of withholding or withdrawing treatment was higher in this study than that previously reported in other European and American studies [3, 22, 38]. This difference might be due to differences in laws, traditions, religious beliefs, or ethical views [22, 39]. Most of the previous studies are more than 10 years old, and the results from one of them suggest that the incidence of withholding and withdrawal of life-sustaining support from critically ill patients has increased over the past decade [3]. A consequence of the increasing therapeutic possibilities is the possible increase in the incidence of withholding or withdrawing life-sustaining treatments. Other studies have revealed differences in the incidence of treatment restriction between surgical and general ICUs [12] and between ICUs in teaching hospitals and those in community hospitals [6]. In the present study, we found that the incidence of treatment restriction did not differ between medical and surgical patients.

This study was subject to some limitations. This study investigated patients who had been admitted to an ICU unit, and an assessment of the selection of patients to the ICU was beyond the scope of the study. However, one should be aware of this limitation when generalizing the findings to other populations or settings.

We did not record the exact time of imposition of life-sustaining treatment restriction; hence, some patients may have had treatment withheld or withdrawn during the first 24 h of the ICU stay, and it is possible that a large proportion of those with such early restrictions died early and were excluded from the study.

The CCI was scored retrospectively based on information in the medical records at hospital admission prior to the index ICU stay. Moreover, the CCI was assessed by two of the present authors, but the score for each patient was only assessed by one rater. The assessors in the retrospective assessment of CCI from the charts had access to the complete electronic medical record, and were therefore not blinded to any information in the chart. We did not assess the interrater or test–retest reliability of the CCI rating [40].

The condition of patients in ICUs can change rapidly, and their response to treatment may affect any decision to withhold or withdraw treatment. Hence, time-limited (such as, no improvement after 1 week in the ICU) and event-limited (such as, development of multiorgan failure) variables may be associated with life-sustaining treatment restriction. In the present study covariates were not updated after 24 hours in the ICU, because the presence and timing of such events could not be obtained retrospectively in a systematic way.

The study investigated patients who had been admitted to an ICU, and variables for the assessment of patient selection into the ICU were not available. We acknowledge that there is a possibility of collider bias that may influence the findings in the study. Moreover, the selection prior to the ICU stays may differ from other hospitals, which is a limitation when generalizing the findings to other populations or settings.

Statistically significant correlations were found in the multivariable model, but these do not necessarily imply a causal association. Multicollinearity may exist in the multivariable logistic regression model. For example, age was included in the SAPS II model and was entered directly into the regression model, and malignancy and acquired immune deficiency syndrome were registered in both SAPS II and CCI. Age was categorized into six categories in the SAPS II model, and was entered as a continuous variable in our regression model. Spearman’s rank coefficients for the correlations of SAPS II with age and CCI scores were 0.43 and 0.30, respectively, which means that the likelihood of multicollinearity was low.

Clinicians certainly take comorbidity into account when deciding on life-sustaining treatment restrictions in their daily clinical practice. However, a more systematic approach to the registration of comorbidity may provide a better basis for making decisions concerning the withholding or withdrawing of treatment. The CCI score might be a useful tool in this setting.

## Conclusions

In this study we found that older age, greater illness severity after 24 h in the ICU and greater comorbidity at hospital admission were independently associated with life-sustaining treatment restriction. The CCI score contributed additional information independent of the illness severity rating.
